# Simultaneous quantification of 49 elements associated to e-waste in human blood by ICP-MS for routine analysis

**DOI:** 10.1016/j.mex.2017.10.001

**Published:** 2017-10-15

**Authors:** Ana González-Antuña, María Camacho, Luis Alberto Henríquez-Hernández, Luis D. Boada, Maira Almeida-González, Manuel Zumbado, Octavio P. Luzardo

**Affiliations:** a Toxicology Unit, Research Institute of Biomedical and Health Sciences (IUIBS), Universidad de Las Palmas de Gran Canaria, Paseo Blas Cabrera Felipe s/n, 35016 Las Palmas, Spain; b Spanish Biomedical Research Centre in Physiopathology of Obesity and Nutrition (CIBERObn), Paseo Blas Cabrera Felipe s/n, 35016 Las Palmas, Spain

**Keywords:** Quantification of 49 elements related to e-waste in human blood, ICP-MS, Rare earth elements (REE), Trace elements, Whole blood analysis

## Abstract

Health risks concerns related to e-waste contamination are increasing all over the world, and especially in developing countries. We have developed an easy, quick, and robust method for the quantification of 49 elements associated to electronic consumer products and their e-wastes in human blood. An aliquot of blood (130 μL) is simply diluted using an alkaline solution, and the elements are simultaneously quantified at the picogram-per-milliliter level without the need of clean-up steps. The accuracy, precision, linearity and limit of quantification (LOQ) of the method were assessed. Recovery values at concentration levels between 0.010 and 5 ng mL^−1^ were studied. A range of 89–118% and a range of 87–128% for REE and toxic heavy elements was found respectively. The relative standard deviations (RSD) calculated were lower than 8% for the most elements. The limits of quantification (LOQ) were found to be lower than 0.04 ng mL^−1^ for toxic heavy elements and 0.06 ng mL^−1^ for the REE with some few exceptions in both cases. The validity of the proposed methodology was assessed by analyzing a certified human blood reference material with known concentrations of several elements. The proposed method is suitable for routine use in biomonitoring studies.

## Graphical abstract

**Figure:**
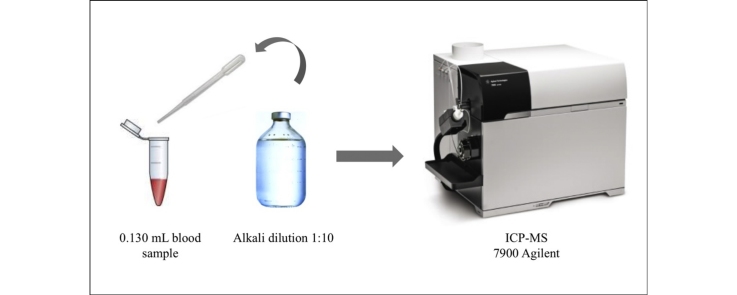

## Description of protocol

### Background information

The intensive use of consumer electronics generates hundreds of thousands of tons of highly polluting waste (electronic waste) all over the world [1]. In the manufacture of these devices, many rare earth elements and minor minerals are used, and as a consequence those elements are appearing as emerging pollutants in many areas of the planet [2]. Pollution is particularly important in developing countries where the processing of electronic waste is done informally and openly on many occasions [3]. There is increasing evidence that these elements can harm the health of humans and other vertebrates [4], and it is becoming increasingly necessary to include these rare elements in biomonitoring protocols [5], [6]. Currently, there are few ICP-MS validated methodologies which allows to simultaneously analyze heavy metals and rare earth elements in human blood. For this reason, in this work, a fast, simple, and robust method has been developed and validated for the determination of the main elements associated with electronic waste in human blood.

### Reagents

A pure standard solution (5% HNO_3_, 100 mg/L) of Ag (silver); As (arsenic); Au (gold); Ba (barium); Be (beryllium); Cd (cadmium); Ce Co (cobalt); Cr (chromium); Cu (Copper), Fe (iron); Hg (mercury); Mn (manganese); Mo (molybdenum); Ni (nickel); Pb (lead); Sb (antimony); Se (selenium); Th (thorium); Tl (tallium); U (uranium); V (vanadium) and Zn (zinc) were purchased from CPAchem (Satara Zagora, Bulgaria). Pure standard solutions of Au (gold, 2% HCl); Bi (bismuth, 2% HNO_3_); Ce (cerium, 2% HNO_3_); Dy (dysprosium, 2% HNO_3_); Er (erbium, 2% HNO_3_); Eu (europium, 2% HNO_3_); Ga (gallium, 2% HNO_3_); Gd (gadolinium, 2% HNO_3_); Ho (holmium, 2% HNO_3_); In (indium, 2% HNO_3_); La (lanthanum, 2% HNO_3_); Lu (lutetium, 2% HNO_3_); Nb (niobium, 2% HNO_3_+ 0.5% HF); Nd (neodymium, 2% HNO_3_); Os (osmium, 10% HCl); Pd (palladium, 5% HCl); Pr (praseodymium); Pt (platinum, 5% HCl); Ru (ruthenium, 2% HCl); Sm (samarium, 2% HNO_3_); Sn (tin, 2% HNO_3_+ 0.5% HF); Sr (strontium, 1% HNO_3_); Ta (tantalum, 2% HNO_3_+ 0.5% HF); Tb (terbium, 2%HNO_3_); Ti (titanium, 2% HNO_3_+ 0.5% HF); Tm (thulium, 2% HNO_3_); Y (yttrium, 2% HNO_3_) and Yb (ytterbium, 2% HNO_3_) were purchased from High Purity Standards (USA). The internal standard solution in 5% of HNO_3_ with Ge (germanium); Ir (iridium); Sc (scandium) and Rh (rhodium) were obtained from ISC-science (Oviedo, Spain). Ultra-pure water (18 MΩ cm^−1^) was obtained from a Milli-Q system (Millipore, Bedford, USA). Hydrochloric acid (30% v/v), nitric acid (65% v/v) and ammonia solution (25% v/v) were bought from Merk (Darmstadt, Germany). All acids and alkaline solutions employed were suprapur grade. Triton X, EDTA (Ethylenediaminetetraacetic acid) and butanol were supplied also by Merk. ClinCal whole blood calibrator was purchased from Recipe Chemicals + Instruments GmbH (Munich, Germany).

### Instrumentation

An Agilent 7900 ICP-MS (Agilent Technologies, Tokyo, Japan) equipped with standard nickel cones, Ultra High Matrix Introduction (UHMI) system and a cross-flow nebulizer with a Make Up Gas Port (X400 Nebulizer, Savillex Corporation, MN, USA) was used for all measurements. UHMI maximizes the plasma robustness of the 7900 ICP-MS through a combination of aerosol dilution and automated optimization of plasma temperature.

The 4th generation Octopole Reaction System (ORS) was able to measure all elements in helium (He) mode, even though, the low-mass elements which are normally measured in no gas mode due to the lack of interferences [7]. The high sensitivity of the 7900 ICP-MS allowed that all measurements were performed in He mode to maximize sample throughput, as was the case in this study.

The optimization of ICP-MS was carried out using a tuning solution consisting in a mixture of Cs (cesium, 55), Co (cobalt, 27), Li (lithium, 3), Mg (magnesium, 12), Tl (thallium, 81), and Y (yttrium, 39) (Agilent Technologies, Palo Alto, CA, USA). All measurements were performed in triplicate from each vial.

The instrument parameters are described in Table 1. The full data was recorded with Agilent MassHunter Data Acquisition software (version 4.2) and processed with Agilent MassHunter Data Analysis software (version 4.2).

**Table 1:** Parameters set by the method in the ICP-MS.

Operating Condition	Values
Sample depth (mm)	10
ORS mode	He
Collision gas He flow (L min^−1^)	5.0
Nebulizer carrier gas flow (L min^−1^)	1.09
Ext 1 lens (V)	0
Ext 2 lens (V)	−90
RF power (W)	1550
RF matching (W)	1.80
Detection mode	Spectral
Integration time (s)	0.3
Repetitions	3

### Procedures

#### Solutions

The alkaline solution were prepared adding 1-butanol (2% v/v), EDTA (0.05% v/v), Triton C-X-100 (0.05% v/v), ammonium hydroxide (1% v/v) and water.

#### Standard solutions

Two multi-element mixtures at 1 μg mL^−1^ were prepared in alkaline solution: A) Ag, As, Au, Ba, Be, Bi, Cd, Co, Cr, Cu, Fe, Hg, Mn, Mo, Ni, Os, Pb, Pd, Pt, Ru, Sb, Se, Sn, Sr, Ti, Tl, Th, U, V, Zn in 2% HNO_3_ and B) Ce, Dy, Er, Eu, Ga, Gd, Ho, In, La, Lu, Nb, Nd, Pr, Sm, Ta, Tb, Tm, Y, Yb. These mixtures were employed to prepare daily diluted calibration solutions.

#### Calibration curves

Two calibration curves from 0 ng mL^−1^ to 300 ng mL^−1^ were prepared using the multi-element mixtures A and B described above with thirty-one elements and nineteen elements respectively [8]. The internal standard, a mixture of Ge, Ir, Rh and Sc, were added to all calibration points and samples to give a final concentration of 37 ng mL^−1^.

#### Sample preparation

0.130 mL of sample was diluted by a factor of ten using the alkaline solution [9]. Then, the internal standard was added and the mixture was shaken. The blanks consisted of 0.130 mL of alkaline solution and the same amount of the internal standard was added.

#### Recovery studies

Recovery studies were performed without any blood matrix. They were performed in alkaline solution due to the commercial lack of a whole blood free of the target elements. For this purpose, the alkaline solution was fortified at a levels of 0.1, 0.5 and 5 ng mL^−1^ and at level of 0.01, 0.1 ng mL^−1^ using the mixtures A and B, respectively. Then, the recovery values were calculated dividing the obtained concentration by the theoretical concentration calculated from the added amount of the solution standard A and B. Three independent replicates of each concentration were prepared, and each one of them analyzed by triplicate.

#### Limit of quantification (LOQ)

The LOQ of the proposed methodology were calculated by quantifying the blank values in ten independent replicates of 0.130 mL of alkaline solution following the sample preparation protocol describe above. For this purpose, the corresponding amount of internal standard was added in all blanks. The LOQ were calculated as three times the concentration of the blanks for all elements.

## Validation

### Calibration curves and limit of quantification (LOQ)

Two calibration curves were performed in order to avoid the interferences of doubly charge ions from some rare earth elements, such the case of Nd and Sm (double charge ions with an *m*/*z* ratio from 71 to 75) which interfere with Ge and As. For the same reason, Ge was removed as internal standard with rare earth elements calibration curve. Good linearity (regression coefficients >0.998) were observed for all elements analyzed by ICP-MS.

The LOQ for 0.130 mL of sample volume was found to be lower than 0.4 ng mL^−1^ and 0.06 ng mL^−1^ for toxic heavy elements and rare earth elements, respectively (Table 2). Only for Ti, Fe, Cu, Zn Sr, Sn and Ga higher LOQ were observed (Table 2). According to these results the proposed methodology can be efficiently applied for the quantification of trace elements in whole blood for biomonitoring analysis, and the obtained results can be fully compared to those reported in the bibliography [10], [11].

**Table 2:** Limit of Quantification in ng mL^−1^ (LOQ) obtained for ten independent replicates. LOQ has been calculated as three times the concentration of the blank solution.

Element	LOQ (ng mL^−1^)	Element	LOQ (ng mL^−1^)
Be	0.03	Tl	0.03
Ti	1.0	Pb	0.07
V	0.07	Bi	0.01
Cr	0.5	Th	0.03
Mn	0.3	U	0.02
Fe	4.0	Ga	0.14
Co	0.05	Y	0.02
Ni	0.3	Nb	0.01
Cu	1.6	In	0.05
Zn	8.9	La	0.06
As	0.02	Ce	0.04
Se	0.4	Pr	0.02
Sr	0.9	Nd	0.03
Mo	0.05	Sm	0.01
Ru	0.05	Eu	0.01
Pd	0.02	Gd	0.01
Ag	0.03	Tb	0.01
Cd	0.01	Dy	0.01
Sn	0.7	Ho	0.01
Sb	0.1	Er	0.01
Ba	0.4	Tm	0.01
Os	0.1	Yb	0.01
Pt	0.02	Lu	0.01
Au	0.04	Ta	0.02
Hg	0.1		

### Accuracy and precision

The accuracy and precision of the proposed methodology was assessed performing recovery studies using alkaline solution fortified at three different levels of concentration, as previously described. The sample preparation was emulated by diluting with the same alkaline solution in the same way described above for the samples (1:10 v/v).

**Fig. 1:**
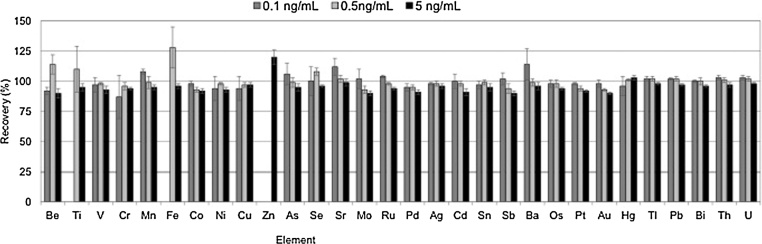
Recovery studies in fortified alkaline solution at 0.1, 0.5 and 5 ng mL ^−1^ for 31 elements. Uncertainty is expressed as the standard deviation of n = 3 independent replicates. (Grey 0.1 ng mL^−1^, pale grey 0.5 ng mL^−1^ and black 5 ng mL^−1^).

**Fig. 2:**
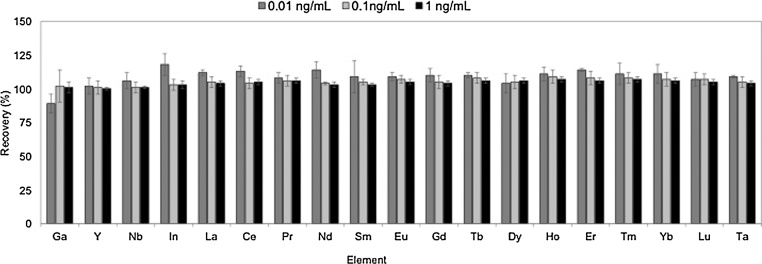
Results of the recovery studies in fortified alkaline solution at 0.01, 0.1 and 1 ng mL^−1^. Uncertainty is expressed as the standard deviation of n = 3 independent replicates. (Grey 0.01 ng mL^−1^, pale grey 0.1 ng mL^−1^ and black 1 ng mL^−1^).

Fig. 1, Fig. 2 show the recoveries obtained, which ranged from 89 to 128% for REE, and from 87 to 118% for toxic heavy elements. In general, the calculated relative standard deviations (RSD) were lower than 8%. However, for some elements (Ti, Cr, Cu, Ni, Se, Fe, Ba, Zn, Sm), the RSD raised to 15–16% at the at the lowest level of fortification. On the other hand, the precision was improved at the highest level of concentration studies, as it was lower than 5% for all elements.

### Assessment of the methodology

The proposed methodology was assessed by analyzing aliquots of a human blood material with a known concentration of several elements (ClinCal whole blood calibrator, RECIPE Chemicals + Instruments GmbH, Munich, Germany). Two dilution factors, 1:10 and 1:20, were carried out in order to study the effect of the blood matrix. Three independent replicates were taken from the same blood vial and three blood vials were also analyzed. All samples were analyzed by a triplicate each one. The results obtained are given in Table 3. As it can be seen, the accuracy for As and Se was improved when a higher dilution was selected (1:20 vs. 1:10). The reason behind this may be the ionization of these compounds could be affect by the whole blood matrix. However, as shown in Table 3 the accuracy for the rest 47 elements was good at both dilution levels, and the calculated concentrations were in agreement with the certified values for all elements reported. The RSD values (%) obtained for three independent replicates of the material were lower than 5% except for As, Hg and Se. Our results show that this method is valid for quantification of these elements in human whole blood samples without the need of additional clean-up steps, and without appreciable matrix effects for all elements.

**Table 3:** Results obtained in the analysis of the ClinCal- whole Blood Calibrator (human blood) by ICP-MS. (a) samples diluted 1:10. (b) Samples diluted 1:20.

Element	Theoretical (ng mL^−1^)	Accuracy^a^ (%)	RSD^a^	Accuracy^b^ (%)	RSD^b^
As	24.1	142 ± 7	5	118 ± 9	7
Cd	8.7	106 ± 5	5	103 ± 3	3
Cr	12.9	108 ± 2	2	102 ± 3	3
Co	15.5	84 ± 3	4	88 ± 4	4
Cu	1960	103 ± 4	3	94 ± 5	5
Pb	357	72 ± 3	4	71 ± 3	4
Mn	24.7	82 ± 3	3	82 ± 3	3
Hg	8.45	104 ± 6	6	84 ± 10	12
Ni	14.9	91 ± 3	4	94 ± 5	5
Pd	4.6	94 ± 3	3	95 ± 4	5
Pt	6.44	105 ± 5	5	102 ± 5	5
Se	187	133 ± 6	5	113 ± 7	6
Ag	9.55	82 ± 3	4	85 ± 4	5
Tl	12.9	72 ± 3	4	72 ± 3	4
Zn	8460	124 ± 4	3	109 ± 6	5
